# Inversion of the Uterus

**Published:** 1879-02

**Authors:** H. P. C. Wilson

**Affiliations:** Baltimore, Maryland


### INVERSION OF THE UTERUS.

By H. P. C. WILSON, M.D., Baltimore, Maryland.

I desire to place upon the record the following case of inversion of the uterus as a tribute to the statistics of this important subject. This misplacement is rare, is often overlooked, when discovered is often neglected as beyond the power of the practitioner to rectify, and is sometimes included in the term "falling of the womb," which, under the ignorance of the past or inattention or thoughtlessness of the present, embraces all forms of malposition of the uterus.

Mrs. E. B. was nineteen years old in April, and married in December, 1871; was confined with her first child September 15, 1876, after five hours of natural labor. The child was living and healthy, but died three months after birth. She conceived again in February, 1877, two months after the death of her child, and was delivered of her second child November 7, 1877, after seven hours of natural labor. She nursed this child two months after delivery, and then weaned him because of her great debility. She is of very fair complexion, lax muscular fibre, light hair and blue eyes.

Her physician was one of the most eminent practitioners of the eastern shore of Maryland. He told me that he never attended a more natural or easier labor. A few minutes after the child was born, he placed one hand on the abdomen, and made very moderate pressure on the uterus, while he tightened the cord gently with the other hand. The after-birth came away with a sudden splash, and a mass (which he took to be a procidented uterus) protruded at the same moment about two inches from the vulva. He pushed it back at once. It re-entered the pelvis without any difficulty, and he saw, heard and thought no more of it.

I saw Mrs. B. for the first time May 3, 1878. She was then perfectly anæmic, and so feeble that she was obliged to be on the bed almost constantly. Her countenance was dejected, and she was spiritless and hopeless. Since the birth of her child (six months ago) she has been losing blood from the uterus constantly, and at times profusely, with dragging sensations about the hips, back, and lower abdomen; yet no vaginal examination had been made to discover the condition of the uterus, and she was sent to Baltimore on a visit to friends, with the expectation that change of scene, air, diet, and association would overcome these symptoms, and restore her to health and strength.

It was the day after she arrived that I first saw her, and a few minutes' conversation satisfied me that there was something radically wrong about the uterus, yet she stoutly resisted my making a vaginal examination, being convinced in her mind that there was nothing wrong in that quarter and insisting she was only weak; and it was not until I was about leaving the room with the announcement, "I had rather give up the case than prescribe without knowing what was the matter," that she very reluctantly consented to a digital examination only.

The index finger of the left hand, on entering the vagina, came in contact with a tumor, the lower end of which was just within the vulva, and the upper end apparently projecting from a dilated os uteri by a neck or pedicle. The tumor was about two and three-fourths inches in its long diameter, and one and a half inches in its greatest transverse diameter. It was dense, and in every particular resembled a fibroid polypus coming from the cavity of the uterus. The pedicle was completely encircled by a dilated os, and in a moment after my finger was inserted into the vagina, I was on the point of announcing the presence of a pediculated fibroid tumor of the cavity of the uterus.

In a large experience in diseases of women for twenty-eight years, I had never before seen a case of inversion of the uterus (either acute or chronic), and so perfectly did the tumor before me simulate a fibroid that I was within an ace of being misled in my diagnosis, and of advising an operation for the removal of a fibroid. These mistakes have often been committed in similar cases, and the uterus removed by mistake; but they ought never to occur, when all the means of diagnosis are employed, and we are careful not to jump at conclusions.

The impression of a fibroid tumor had scarcely flashed across my mind, when it was replaced by the recollection that this lady was in perfect health up to the time of her last confinement, and had not had a well day since; so that her present condition must in some way be connected with that occasion. I began to feel by bi-palpitation for the body of the uterus. It could nowhere be found, either per rectum or per vaginam, and I decided at once that the tumor before me must be the uterus. To make my diagnosis certain, I placed my patient in Sims' left lateral position, introduced the speculum, and attempted to pass the sound by the side of the apparent tumor, through the dilated os, into the cavity of the uterus; but all my efforts failed. These digital and probing examinations settled me in my opinion, and I at once announced to the patient that she had inversion of the uterus, and would require an operation for its restoration. I desired her to write at once to her physician of my diagnosis, and to ask that he would be present at the operation.

The time for the return of her menses was at hand.

They were just coming on, and I advised a return to her home on the eastern shore, where, with her husband, child and friends, she would have less of the terrors of an operation hanging over her than by remaining in Baltimore. This she did, and came back to me, with her physician, four or five days after menstruation had ceased.

I may remark here in passing that I saw in this case what I have never seen before, and shall probably never see again—the process of menstruation going on from the surface of the uterus turned inside out. It gave the impression of a sweating of blood from the surface of an engorged mucous membrane, just like the sweating of perspiration from the surface of the skin over an excited capillary circulation.

On the 17th of May, thirteen days after I first saw her, and five days after menstruation had ceased, after she had been examined by her physician and my diagnosis verified, she was given a liberal drink of whisky and then chloroformed to complete relaxation, by Dr. Gardner. Lying on a table in the dorsal position, with thighs flexed on abdomen and legs flexed on thighs, one knee steadied by Dr. Bayley and the other by a nurse, having first pared closely my finger-nails, I proceeded to the reduction of the inversion.

One hand was passed completely into the vagina, and, the fundus uteri resting in its palm, the neck was encircled by the fingers, and steady upward pressure was made against that portion of the uterus which last emerged from the external os, while the other hand made steady counter-pressure above the pubis. The fingers were separated as far as possible from time to time to expand the encircling os, and allow the neck and body to return more easily. My plan was to return first the portion last inverted, until the fundus should disappear through the internal os.

At the end of half an hour of steady pressure, first with one hand and then with the other, I had succeeded in reduction to the point of bringing the lower end of the fundus within the external os, but all efforts to carry the body through the internal os were unavailing for some time longer. My fingers became so cramped, and my hands and arms so powerless, that I was obliged to desist from time to time, and replace my hands with those of Dr. Gardner, who rendered the most valuable assistance in every step of this operation.

When I had reached that point in reduction where the fundus had entered the external os, and all efforts to advance it through the internal os were unavailing, I changed my plan of attack. I indented the fundus uteri with the index-finger of one hand, and made counter-pressure with the index-finger of the other hand, pressing firmly down into the internal os from above the pubis; but all efforts in this direction failed.

I then attempted by indenting first one horn of the uterus and then the other, while the same counter-pressure was made as above, but with no more success. I then returned to my first manipulation, of grasping the fundus with my hand, and the cervix with my fingers, and making steady pressure upward against steady pressure downward from above the pubis, and at the end of one hour and ten minutes from the commencement of the operation we were rewarded with complete reduction of the inverted uterus.

My fingers, hands and arms were almost powerless at the end of the operation, and I should have failed in the reduction at this first attempt, but for the aid given me by Dr. Gardner. The extent of this paralysis may be appreciated, when I state that I was unable to use my pen or perform any delicate manipulation for several days. I have never experienced such paralysis of the hand or arm in any previous operation within the pelvis.

The chloroform in this case was most skillfully administered. She took in all eight ounces, and was kept perfectly relaxed from beginning to end.

After the reduction the uterus was mopped out with Monsell's solution of sub-sulphate of iron and glycerine as an antiseptic. A pledget of cotton soaked in glycerine was placed in the vagina against the os, and the patient lifted into bed. She received no other treatment but plenty of milk and a liberal diet, was kept in bed four or five days, and had her uterus mopped out every other day for ten days, first with the above solution of iron and glycerine, and then with Churchill's caustic iodine. At the end of this time she was allowed to return to her home with no other directions than to live liberally, drink plenty of milk, and wash out the vagina once daily with very hot water. She returned to see me in six weeks, looking well, healthy, happy, and full of life and gratitude.

Just before she left for home I noticed that the uterus was inclined to fall backward, and in her relaxed, anæmic state, with all its natural supports exhausted, and stretched to their fullest capacity, I deemed it best to insert a small Hodge's pessary, rather than run the risk of complete retroversion. This was done with great comfort to the patient.

As stated in the commencement of this paper, this is the first case of inversion of the uterus I have ever seen; and, to give some idea of how rarely it occurs, it was "observed at the Rotunda Hospital but once in upward of 190,800 deliveries," in a period of over thirty years. It most commonly occurs immediately after labor by pulling on the cord while the placenta is still attached to the walls of the uterus; and when it thus occurs, if recognized at once, it is very easily reduced by pressing it immediately back through the relaxed and dilated os uteri. Every day, month or year that it remains unreduced, its reduction becomes more difficult, and after great length of time often impossible.

Inversion of the uterus is sometimes produced immediately on expulsion of the child, where there has existed an unusually short funis, and this wrapped several times around the child's neck. The weight of the child under such circumstances may pull the fundus through the external os, by dragging on an insufficiently lengthy cord. It is as easily reduced as in the previous case, if discovered at once. Or inversion of the uterus may occur immediately after labor, where there has been no pulling on the cord, by the weight of an attached placenta dragging the uterus through the dilated os. It is as easily rectified in this as in the previous cases, if observed and undertaken at once.

Inversion of the uterus may also occur soon after labor, where the placenta has neither been pulled upon nor has its weight dragged the body of the uterus through the dilated os. It may take place in an anæmic woman of lax muscular fiber, where there are irregular and partial contractions of the body of the uterus, by which the semi-paralyzed seat of placental attachment is forced through the dilated os by other portions of the uterus contracting around it. Inversion occurring from these causes is not susceptible of as easy reduction as in the previous cases mentioned; but, if promptly undertaken, and, if necessary, calling in the aid of chloroform, there is usually no great difficulty in replacing the uterus.

To this class of cases belongs the very remarkable one recently reported by my friend Dr. Byrne, of Brooklyn, in the New York *Medical Journal* for October, 1878, and which he styles "unavoidable or spontaneous" inversion. In this case the hand carried into the cavity of the uterus immediately after the delivery of the placenta (which was found in the vagina) encountered a partially inverted fundus. This inversion was readily reduced by upward pressure with the fingers, but invariably returned on withdrawal of the hand; and, as he states there was "no active hemorrhage," in all probability the uterus was well contracted around this semi-paralyzed fundus (no doubt the recent seat of placental attachment), and thus the fundus was forced into "unavoidable inversion." Notwithstanding the skillful manipulation of this distinguished gynæcologist, he was unable to prevent this partial inversion from becoming a complete one. The entire body of the uterus passed through the cervix into the vagina, and all justifiable manipulation failed in its replacement till nine days after its occurrence.

This is an exception to the general rule that these cases are easily reduced if promptly discovered. I refer the reader to Dr. Byrne's paper for this interesting case of inversion as well as for the ingenious instrument invented to replace it.

Sometimes inversion of the uterus is produced by a fibroid tumor in its cavity dilating the cervical canal, and then by its weight, dragging tne body of the uterus through the external os into the vagina. The tumor should be removed and the reduction undertaken at once.

To this class of cases belongs the interesting one reported by Dr. T. Gaillard Thomas, in the October number of the *American Journal of Obstetrics* for 1878, in which a fibroid tumor was the cause of complete inversion of both uterus and vagina. Tumor, uterus and vagina appeared as one mass without the vulva. The woman had not been pregnant for thirteen years, and the condition in which he found her had existed for three or four years.

Another cause of inversion of the uterus is too great pressure through the abdominal walls on the fundus of a relaxed uterus. In this way the fundus may be indented, and very little irregular uterine contractions may be sufficient to carry on the work, till the fundus emerges through the external os.

In this, as in all other cases, inversion of the uterus is usually reduced with ease, if recognized early and undertaken at once. Those of months' and years' standing are the ones that give the practitioner so much trouble, and sometimes prove entirely beyond his control; and hence the importance of seeing, immediately after every labor, that the uterus is in proper place and condition.

Injudicious pulling on the funis by the *accoucheur* is the cause of more cases of inversion of the uterus than all other causes combined. It has been my habit in obstetrical practice, for many years, never to tighten the cord, unless an examination with the index-finger discovered the placenta in the vagina. Then there is no objection to pulling it away by the cord. But, if the placenta remains in the uterus after the cord has been tied and the child handed to the nurse, I immediately grease my hand and pass it into the uterus. If the placenta is detached, I turn it out with the hand, just as I would turn out a mass of clotted blood; if it is attached, I peel it off with the finger nails, turns it out, and manipulate the uterine cavity till contractions expel my hand. I thus secure firm contraction of the uterus, seldom encounter *post-partum* hemorrhage, and diminish the chances of septicæmia, by more effectually closing the mouths of all open vessels, and more thoroughly cleansing the cavity of the uterus. I also secure the patient against inversion, and lessen many of the other dangers to which parturient women are liable.

I am aware that at least one of my most distinguished friends, Dr. Fordyce Barker (whose teachings I delight to treasure, and whose warnings should never go unheeded), cautions against the introduction of the hand into the cavity of the uterus after labor, and thinks it is fraught with the danger of lacerating the cervix; but I cannot see how the cervix uteri can escape laceration while the head and shoulders of a child are passing through it, and meet with it by the introduction of the hand immediately after delivery.

It must be a very large hand, and very rough manipulation, that could produce such a result immediately after expulsion of the child; and, when laceration has followed such a manual exploration, I would think it due rather to the egress of the child than the ingress of the hand.

I consider this use of the hand free from all danger. We gain thereby perfect intelligence of the condition of the cavity of the uterus, and secure, as by no other means, firm and permanent contraction of the same. We are cognizant at once of threatened inversion, threatened hemorrhage, threatened hour-glass contraction, adherent placenta, and any remaining *deciduæ*, and thus have the knowledge of any impending danger, as well as remedy, at our fingers' ends.

